# Change in modifiable maternal characteristics and behaviours between consecutive pregnancies and offspring adiposity: A systematic review

**DOI:** 10.1111/obr.13048

**Published:** 2020-05-29

**Authors:** Elizabeth J. Taylor, Sam Wilding, Nida Ziauddeen, Keith M. Godfrey, Ann Berrington, Nisreen A. Alwan

**Affiliations:** 1 School of Primary Care, Population Sciences and Medical Education, Faculty of Medicine, University of Southampton, Southampton, UK; 2 MRC Lifecourse Epidemiology Unit, University of Southampton, Southampton General Hospital, Southampton, UK; 3 Department of Social Statistics and Demography, University of Southampton, Southampton, UK; 4 NIHR Southampton Biomedical Research Centre, University of Southampton and University Hospital Southampton NHS Foundation Trust, Southampton, UK

**Keywords:** adiposity, childhood, interpregnancy, obesity

## Abstract

Causal evidence links modifiable maternal exposures during the periconceptional period with offspring obesity. The interconception period may be an important time to intervene. We systematically identified studies examining change in modifiable maternal exposures between pregnancies and offspring adiposity. We searched for longitudinal studies published between 1990 and 2019, which included measurements taken on at least two occasions in the period from 1 year prior to the conception of the first birth to the time of the second birth, and which included a measure of adiposity in second, or higher order, siblings. Age, ethnicity and genetics were not considered modifiable; all other factors including length of the interpregnancy interval were. Eleven studies satisfied the inclusion criteria. Higher interpregnancy weight gain or loss, maternal smoking inception, mothers smoking in their first pregnancy and quitting, increasing the number of cigarettes smoked and longer interpregnancy intervals were positively associated with adiposity in second or higher order children. Vaginal birth after caesarean delivery was protective. Further research is needed to ascertain whether the risk of adiposity is fixed based on first pregnancy exposures or if interpregnancy change alters the risk for a subsequent child. This can inform the type and effectiveness of interventions for mothers prior to a subsequent pregnancy.

## 1 Introduction

The World Health Organization estimated that in 2016 there were 41 million children worldwide under the age of 5 and 340 million children and adolescents aged 5–19 who were affected by overweight or obesity.^1^ More than 1.9 billion adults were affected by overweight, and, of these, 650 million (11% of men and 15% of women) were affected by obesity.^1^ Early intervention and prevention is key since childhood weight is strongly associated with adult weight^2^ and obesity raises the risk of many noncommunicable diseases including cardiovascular disease,^3^ Type 2 diabetes,^4^ osteoarthritis,^5^ other musculo-skeletal disorders^6^ and a number of different types of cancer.^1^

The Developmental Origins of Health and Disease (DOHaD) concept links exposures during the periconceptional period and pregnancy to lasting effects on developmental programming of the foetus and to subsequent susceptibility to noncommunicable disease.^7^ A number of modifiable maternal characteristics and behaviours in the preconception, pregnancy and interpregnancy period (defined by the World Health Organization as the period from the birth of one child until the conception of the subsequent pregnancy^8^) have been associated with offspring adiposity.^9–12^ These include, but are not limited to, maternal obesity, with or without maternal gestational diabetes mellitus (GDM),^13^ gestational weight gain,^14^ smoking,^15^ severe pre-pregnancy stress,^16^ birth by caesarean section^17^and inequalities in socioeconomic status.^18,19^

Whilst there is much in the literature examining associations between the exposures in one pregnancy and the outcome for that child, there is little research on whether this risk is then fixed for subsequent children based on those earlier predictors, or whether a change in exposure between pregnancies changes the risk for a subsequent child. The majority of research which has examined inter-pregnancy change, focuses on birth or neonatal outcomes such as large or small-for-gestational-age and premature birth.^20–23^

We aimed to systematically review the literature for longitudinal studies which characterized modifiable maternal exposures between successive live pregnancies and determine their associations with adiposity in second or higher order siblings.

## 2 Methods

### 2.1 Search strategy

An electronic search was conducted using the bibliographic databases EMBASE (via Ovid), MEDLINE (via Ovid), CINAHL (via EBSCO), PsycINFO (via EBSCO) and Web of Science. A specialist librarian was consulted in advance, and the search was run on 30 March 2019. Studies were restricted to those based on human participants and published in English since 1990 to ensure that only the most up-to-date literature was reviewed. The protocol for this review was published on the international prospective register of international reviews, PROSPERO, reference CRD42019124344,^24^ and this review is reported in line with the PRISMA guidelines.^25^ The following search strategy was piloted on EMBASE and adapted for the other platforms:

[exp family planning/] OR ((interpregnan* or inter-pregnan* or consecutive pregnan* or interconception or inter-conception or between pregnan* or family planning or inter-natal or multipar* or multigravid* or conception).mp.)] AND

[exp obesity/ OR exp body mass/OR exp overnutrition/OR ((obes* or overweight or overnutrition or adiposity or weight or body mass). mp.)] AND

[exp progeny/OR exp child/or exp sibling/OR ((child* or offspring or infant or sibling* or progeny).mp.)]

### 2.2 Inclusion and exclusion criteria

We only included longitudinal studies. Case control studies, cross-sectional studies and case reports were excluded.

**Figure 1:**
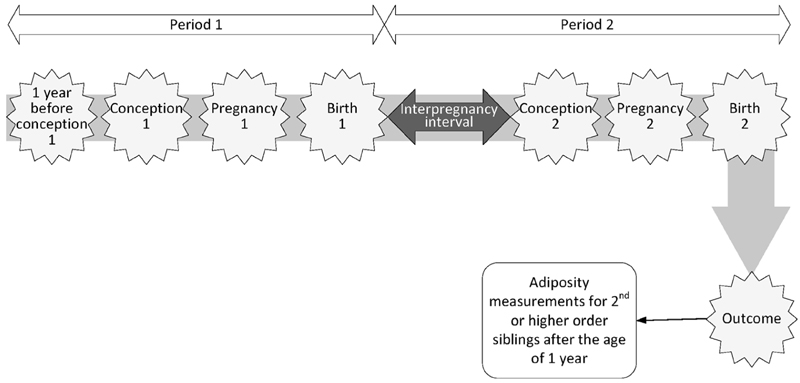
Diagram representing the period of time under consideration in this systematic review. At least one exposure measurement should have been recorded in Period 1 and at least one in period 2. Outcome measurements should be recorded in second, or higher order, siblings after the age of 1 year, without upper bound

We did not consider age, ethnicity or genetics to be modifiable, but considered all other maternal behaviours and characteristics to be potentially modifiable exposures. Studies where measurements of the main exposure were taken on at least two occasions in the period from 1 year prior to the conception of the first pregnancy up to the time of the second birth were considered. At least one measurement should have been recorded in the period from 1 year prior to the conception of the first pregnancy and the first birth (Period 1) and at least one in the period from the time of the first birth to the time of the second birth (Period 2). Figure 1 represents the relevant timeline.

The length of the interpregnancy interval was specifically included as a modifiable exposure as defined per individual study, even if the World Health Organization definition of the inter-pregnancy interval was not used (the period from the birth of one child to the conception of the subsequent pregnancy).^8^

We considered studies where outcomes included a measure of adiposity in second or higher order siblings. Appropriate outcome measurements included categorical and continuous offspring BMI, weight for height *z*-scores, skinfold measurements and body adiposity. All time points were considered after the age of 1, without an upper age limit. The lower bound was given as 1 year to exclude studies focussing on neonatal outcomes, including foetal macrosomia.

### 2.3 Screening process

The screening management software Rayyan^26^ was used to screen titles for eligibility. A randomly selected sample of 10% of these titles was independently screened by two reviewers (EJT and SW). A 10% sample was chosen as a recent simulation study has shown that if the fraction being sampled for independent review is increased, there is no decrease in selection bias.^27^ At the title screening stage, the percentage agreement between the two reviewers was 98.9%, and discrepant titles were included for abstract screening. One author (EJT) reviewed the remaining titles. The same process was then followed for reviewing the abstracts of papers remaining after title screening. The agreement rate between the two reviewers (EJT and SW) was 100%, and accordingly, one author (EJT) reviewed the remaining abstracts.

Following abstract screening, the full texts of the remaining titles were accessed and reviewed by two reviewers (EJT and SW), with one exception where the discrepant title was only considered by one reviewer (EJT), due to copyright restrictions, and was excluded in any event.

### 2.4 Data extraction, quality assessment and analysis

A modified version of the Cochrane Collaboration data extraction form was used to conduct data extraction,^28^ and this was undertaken by two reviewers (EJT and SW or EJT and NZ).

All eligible data were extracted, including for subgroup analysis and exposure multiple time points where these were reported. Where reported, confidence intervals which did not overlap the null were used to identify statistically significant associations. If these where not reported, *p* values were used.

Quality assessment was also undertaken by two reviewers (EJT and SW). Key strengths and weaknesses of each of the cohort studies^29–38^ were formed using the National Heart Lung and Blood Institute assessment tool for observational cohort and cross-sectional studies.^39^ The remaining study is an individual patient data meta-analysis,^40^ and in this instance, we used the PRISMA-IPD checklist.^41^ The overall rating for addressing the risk of bias (good/fair/poor) is included in Table 1.

Since we expected significant variation in study exposures and design, a qualitative synthesis was planned *a priori* rather than a meta-analysis. Studies have been grouped based on the exposures being considered, and we summarize the effect sizes and precision for each included study.

## 3 Results

**Figure 2:**
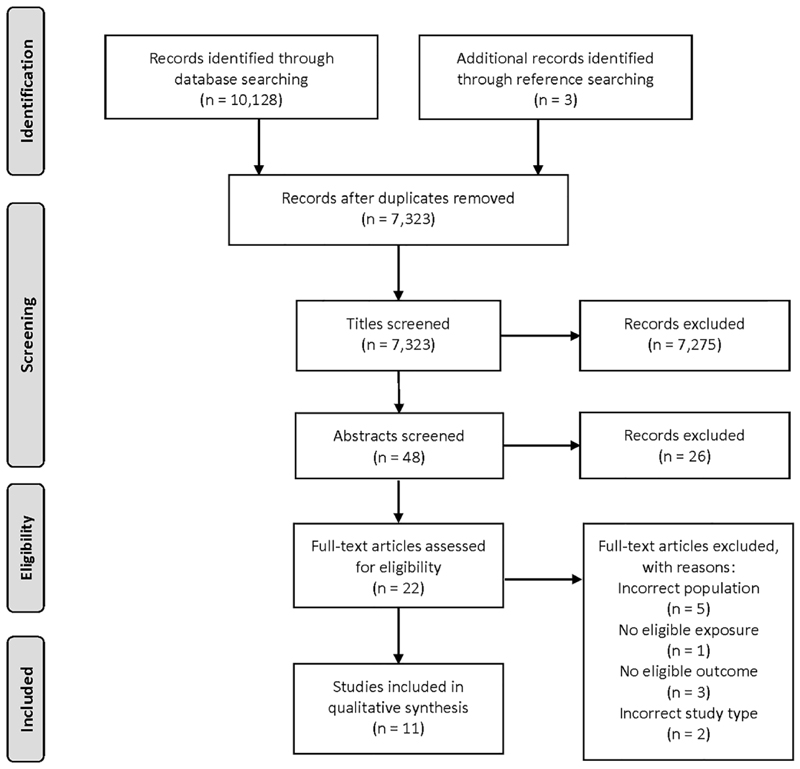
PRISMA flow diagram

All stages of data screening were documented using a PRISMA flow diagram^25^ (Figure 2). Our searches identified 10 131 records of which 2808 were duplicates. A total of 7323 titles were screened and of these, 48 abstracts were screened. Full texts of 22 papers were assessed for inclusion, and of these, 11 have been included in the qualitative synthesis.

Three of the included studies were based on populations in Sweden,^31,34,37^ two each in America,^35,38^ Australia^29,36^ and Brazil^32,33^ and one in Scotland.^30^ The remaining study^40^ used data from five underlying datasets from Australia, Canada and America; one of which was the Collaborative Perinatal Project which was also used by one of the other studies.^35^ Three studies included maternal interpregnancy weight change as the exposure,^29,30,37^ three examined maternal smoking status,^30,34,40^ two considered mode of birth^36,38^ and four the length of the interpregnancy interval.^31–33,35^ Further study characteristics are detailed in Table 1. Associations between change in exposures and the risk of offspring adiposity are summarized in Table 2. A summary of the statistical analysis undertaken, adjustments made and significant results reported in each study is given in Table 3.

### 3.1 Characteristics of the included studies

Table 1 summarizes the included studies. With the exception of the individual patient data meta-analysis,^40^ all included studies use data from cohorts^29,32,33,35,38^ and data-linkage studies.^30,31,34,36,37^ These studies showed considerable variation in terms of the exposure and outcome measurements.

Studies where the exposure was interpregnancy weight change,^29,30,37^ smoking status^30,34,40^ or mode of birth^36,38^ had a measurement taken during Period 1 and a further measurement in Period 2, as defined above (Figure 1). Most studies included only one time-point for outcome measurement and the timings varied by study; the youngest mean age for offspring was 19 months^33^ and the highest 30.2 years.^32^ Both of these studies^32,33^ followed children whose mothers formed part of the 1982 Pelotas (Brazil) Birth Cohort Study.^42^ One study from America followed offspring from ages 9–14 years through ages 20–28 years, considering annual time points for 5 years and biennial time points thereafter.^38^ One study from Sweden used measurements taken at comprehensive health checks when the child was 4 years of age and used these to predict BMI on the child’s actual fourth birthday.^37^ Two studies utilized Swedish conscription data and only included outcome measurements for males.^31,34^

Across the 11 studies, there was one categorical and a number of continuous outcomes. Three studies considered more than one relevant outcome^30,32,37^ (Table 1). For studies other than the meta-analysis,^40^ these outcome measurements were taken by school nurses,^30^ military personnel,^31,34^ research centre staff,^32,33^ clinic staff^35,37^ and nurses.^36^ In one case, measurements were self-reported,^38^ and in another, mothers were provided with measuring tapes and instructions on how to take measurements.^29^

The four maternal exposures which emerged from this review are reviewed in turn below and summarized in Tables 1–3.

### 3.2 Length of the interpregnancy interval

Of the four studies, which considered the length of the interpregnancy interval as the exposure, one was judged to be good,^31^ two fair^32,33^ and one poor^35^ in terms of their risk of bias (Table 1).

Barclay and Kolk^31^ considered the long-term consequences of the length of both preceding and subsequent (not considered here) inter-pregnancy intervals on offspring health. They found that, compared with a birth interval of 25 to 30 months, men born after an interval of at least 31 months had a higher probability of being affected by overweight/obesity in young adulthood. Between-family analysis showed an increasing trend as the interpregnancy interval increased, and within-family analysis showed a broadly similar pattern^31^ (Table 3).

Both Devakumar et al.^32^ and Huttly et al.^33^ followed children whose mothers formed the 1982 Pelotas (Brazil) Birth Cohort Study,^42^ but both studies considered different outcome measurements and time points (Table 1). Huttly et al.^33^ reported mean weight-for height *z*-scores which generally increased with the length of the interval (Table 3). Devakumar et al.^32^ found a positive association for fat-free mass and visceral fat^32^ for female offspring where the birth interval was treated as a continuous variable and a positive association with offspring fat-free mass when the birth interval was treated as a binary variable (Table 3).

In contrast, Li et al.^35^ did not find an association between the length of the interpregnancy period and offspring BMI *z*-score at age 7 years.

These four studies had objectively measured outcomes,^31–33,35^ but in the case of two, the exposure was based on maternal recall.^32,33^ One study was limited to considering shorter birth intervals since the duration from recruitment to the last birth was 6 years.^35^

### 3.3 Maternal weight change

Three studies considered maternal interpregnancy weight change as the exposure and these were all judged to be fair^29,37^ or good^30^ in terms of their risk of bias (Table 1).

Adane et aI.^29^ found twice the odds of being affected by obesity where there was high interpregnancy weight gain (≥4 kg/m^2^) but reported no increase in odds of offspring obesity for interpregnancy weight loss (>1 kg/m^2^), small gain (1 to <2 kg/m^2^) or moderate gain (2 to <4 kg/m^2^)^29^ (Table 3).

Willmer et al.^37^ examined interpregnancy weight loss due to bariatric surgery between pregnancies. They found no association between the differences in BMI in week 10 of the pregnancy prior to, and the pregnancy after, surgery with differences in sibling’s BMI at age 4 years.

The primary focus of the paper by Aucott et al. was maternal smoking, but they also provide results for children being affected by overweight/obesity based on percentage maternal weight change between pregnancies.^30^ They found for a loss of ≥10% (compared with a change of ±3%) the odds of children being affected by overweight or obesity were increased by 30% and 90%, respectively, and that mean BMI *z*-score was also higher for a gain of ≥10% (compared with a change of ±3%)^30^ (Table 3).

The studies by Aucott et al.^30^ and Willmer et al.^37^ utilized routinely collected population level data in Scotland and Sweden respectively, and included objectively measured outcomes, but in the former, maternal and child records were linked in only 44% of cases while in the latter, full details were only available for 71 child–woman triads. All the exposure and outcome measurements in the Adane et al.^29^ study were self-reported, and only 34% of women responded to the survey asking for their children’s details.

### 3.4 Maternal smoking status

Three studies considered maternal smoking status as the exposure and these were judged to be good^30,34^ and fair^40^ in terms of their risk of bias (Table 1).

Aucott et al.^30^ found that siblings born after a mother had started smoking between pregnancies (i.e., she did not smoke in her first pregnancy but did in her second) had higher mean BMI *z*-scores at age 5 than older siblings who were unexposed^30^ (Table 3). BMI *z*-score was also higher in the younger sibling compared to the older sibling where a mother smoked in both pregnancies, but they found no significant difference where a mother quit smoking between pregnancies (i.e., where she was a smoker in the first but not the second pregnancy).^30^

Without sibling analysis, and compared to never smokers, mean offspring BMI *z*-score and the odds of overweight/obesity where mothers started smoking between pregnancies, always smoked or quit between pregnancies were higher^30^ (Table 3).

Iliadou et al.^34^ found increased odds of being affected by overweight only where the mother smoked in both male pregnancies, compared with mothers who did not smoke during either pregnancy^34^ (Table 3). The odds were not increased where a mother smoked only in her first or only in her second pregnancy. They found no effect where the association was evaluated within full and half sibling pairs.^34^

Albers et al.^40^ performed an individual patient data meta-analysis on five studies to analyse differences in the number of cigarettes smoked across successive pregnancies and the dose-dependent relationship with offspring BMI (Table 1). They found that each additional cigarette smoked a day in sibling pregnancies increased offspring BMI *z*-score^40^ (Table 3).

In both the Aucott et al.^30^ and Iliadou et al.^34^ studies, smoking status was self-reported at a single point during pregnancy. Three of the underlying studies used by Albers et al.^40^ had multiple self-reported measures of the numbers of cigarettes smoked during different stages of pregnancy and they used the maximum number at any time point in their analysis.

### 3.5 Mode of birth

Two studies considered mode of birth as the exposure and these were judged to be good^36^ and fair^38^ in terms of their risk of bias (Table 1).

Smithers et al.^36^ examined the association between mothers who had previously given birth by caesarean section and who subsequently went on to give birth vaginally or by elective caesarean with the second child’s BMI. They found no association between elective caesarean section for the subsequent birth and BMI *z*-score.^36^ Those who gave birth to their subsequent child by emergency caesarean, or who had an instrumental birth, were excluded from the analysis^36^ but over 85% of the subsequent deliveries were by caesarean section.

The association between caesarean section birth and the risk of offspring being affected by obesity also formed part of the study undertaken by Yuan et al.^38^ They compared siblings who were discordant in their mode of birth and found that amongst women with a previous caesarean birth, the risk of offspring being affected by obesity after a subsequent vaginal birth was reduced by 31% (compared to a repeat caesarean birth)^38^ (Table 3). For women who had a previous vaginal birth, the estimated risk of offspring being affected by obesity for a successive caesarean birth was not significant.^38^ Their study does not make a distinction between emergency and elective caesarean sections and includes no data on intrapartum indicators of caesarean birth, or any detailed labour/birth information.^38^

## 4 Discussion

This systematic review included eleven studies that assessed maternal interpregnancy weight change, differences in maternal smoking status, changes in mode of birth and the length of the interpregnancy interval between successive pregnancies in relation to adiposity in second or higher order siblings from age one onwards. Higher interpregnancy weight gain or loss, starting smoking between pregnancies, smoking at the first pregnancy only, or an increase in the number of cigarettes smoked during successive pregnancies and longer interpregnancy intervals showed a positive association. Vaginal birth after caesarean had a protective effect. To our knowledge, this is the first time that evidence on this subject has been systematically reviewed.

The quality assessment and overall rating for addressing the risk of bias has been judged to be either good or fair for all but one of the included studies (Table 1). The study judged as poor reported no results of significance.^35^ There was much variability in the confounders controlled for in the different analyses (Table 3). All but one^40^ of the included studies control for maternal age and the majority for maternal smoking^29,35–38^ and maternal weight in some form,^29,30,32,34,35,38,40^ where these were not the exposures under consideration. Most studies included at least one socio-economic factor.^13,29,30,32–36,38,40^ Only a minority of studies were able to take account of exposures in pregnancy such as diabetes and hypertension.^35,36,38^ Only one study included any adjustment for paternal confounders.^34^ Most studies did not specifically state which pregnancy the confounders related to.

A number of studies have made adjustments for covariates which are potential mediators between the exposure and offspring adiposity. Examples of such adjustments made in the included studies are breastfeeding,^40^ gestational weight gain^34^ and birthweight^30,34–36,38^ and both of the included studies which consider mode of birth adjust for birthweight in some form^36,38^ even though this is a potential mediator (Table 3). Children exposed to diabetes (including gestational diabetes mellitus) in utero are at high risk of childhood adiposity, although breastfeeding has been shown to attenuate this risk.^43,44^ Gestational weight gain has been shown to mediate the relationship between a mother’s BMI and offspring adiposity.^45^ Misclassification of confounders and mediators affects the magnitude of the total effect of the exposure on the outcome;^46^ both the direct effect, which is not through the mediator, and the indirect effect, through the mediator.^47^ Over-adjustment bias can affect the direct effect of the strength of associations and arises when controlling for variables which are on the causal pathway between exposure and outcome.^48^ Unnecessary adjustments of other variables on the causal pathway can affect the precision.^47,48^

All the studies were based on data from high-income or upper middle income countries, which will have had an effect on the prevalence of adiposity^49^ and individual risk factors, including the level of antenatal care,^50^ rates of birth by caesarean section^51^ and smoking.^52^ This limits our ability to generalize the findings to lower income countries. In addition, potential biases may have arisen because many of the included studies had low response rates to questionnaires or surveys asking for details about children,^29^ low rates of data linkage^30,36,53^ or only included male outcomes.^31,34^ Further, the data used in one study were relatively old, with only 6 years from recruitment to the final birth and with final outcome measurements taken in 1972.^35^

Most of the exposure and outcome measurements were objectively recorded by professionals, but where these were self-reported, for example, in the case of smoking status in pregnancy, potential information bias due to under-reporting^54^ may have arisen. Longitudinal cohort studies which use population level data and are linked to objectively measured child outcomes are needed to avoid bias in recruitment and in measurement. A number of the included studies have low response or linkage rates^29,30,36^ or only have details available for very small numbers of participants.^37^

This review has a number of strengths. The initial search was adapted and run through a variety of different online databases. It is unlikely that this search was too narrow since a very large number of studies were retrieved for consideration (Figure 2). Our search was, however, limited to studies published in English, and it is possible that there may be literature published in other languages which could have formed part of this review.

We did not include grey literature in our search, and there may therefore be an element of publication bias towards statistically significant associations.^55^ This does not appear to have been the case, as although most of the studies reported at least one non-null finding, a number only reported nonsignificant associations^34–37^ (Table 2).

Agreement between two authors (EJT and SW) at the screening stage of a random 10% sample of titles and abstracts was very high (98.9% and 100%), and two reviewers considered the full texts of studies remaining after abstract screening (EJT and SW). Data extraction and risk of bias assessments were undertaken by two authors (EJT and SW or EJT and NZ). We then followed a recent approach and listed strengths and weaknesses for each study using two commonly used checklists.^18^ This allowed identification of important criteria in each study.

There are a number of exposures, which are known to have an impact on offspring adiposity, which were not found in the literature searched for this review, for example, gestational weight gain,^14^ maternal prepregnancy stress,^16^ supplement intake and lower maternal vitamin D status,^56^ and measures of social and economic status such as education and area of residence.^18^ Further research into changes in exposures and into combinations and interactions between exposures and the association with the magnitude of the risk of children being affected by overweight/obesity is recommended. We specifically recommend studies which examine differences in vitamin D, folic acid and other vitamin supplementation and those which consider different levels of gestational weight gain. We do not know whether a change in exposure between pregnancies modifies the risk of adiposity for a second or subsequent child or whether the risk is based on past exposures. The World Health Organization recommends a birth to conception interval of at least 24 months in order to avoid a range of poor maternal, perinatal, neonatal and birth outcomes which are particularly associated with intervals under 18 months.^8^ A number of included studies have found associations between the longest interpregnancy intervals and childhood adiposity, and research which combines the effect of the length of the preceding birth interval with other changes in maternal exposures is also recommended.

## 5 Conclusion

Change in a number of exposures across successive pregnancies showed associations with greater adiposity in second or higher order siblings; substantial interpregnancy weight gain or loss, smoking in the first or second pregnancy only, or increasing the number of cigarettes smoked and longer interpregnancy intervals. Vaginal birth after a caesarean birth had a protective effect. There was considerable heterogeneity in the quality and methodology of the included studies.

Further research into changes in modifiable maternal exposures and behaviours between successive pregnancies is recommended to see if the risk of adiposity for second or subsequent children is fixed based on earlier predictors or if this risk changes as exposures and behaviours change.

## Figures and Tables

**Table 1:** Included study characteristics

First author and year	Study design, location and sample size	Maternal exposure(s)	Relevant child outcome(s) and age(s)	Quality assessment and overall rating for addressing the risk of bias (good/fair/poor)^a^
Adane 2018^29^	Population-based prospective cohort study Australia *n* = 714 sibling pairs of children	Interpregnancy BMI change^b^ Calculated by taking BMI measures prior to the conception of each child and categorized: Stable, small gain, moderate gain, high gain	BMI categories based on age and sex-specific cut-offs for children^b^ Second born children aged between 2 and 13 years	Positive Adjusts for maternal confounders Negative All exposure and outcome measures self-reported Lack of data on gestational age and gestational age gain Only 34% of women invited responded to the survey about their children Rating: Fair
Albers 2018^40^	Individual patient data meta-analysis of 5 datasets from studies reporting BMI of children and the number of cigarettes smoked in pregnancy and then scanned for sibling data America (×3) Australia Canada *n* = 45 299 children from 14 231 families	Smoking status Number of cigarettes smoked across pregnancies ^b^ The number of siblings per family ranged from 2 to 16 children. There were two children for *n* = 10 576 families	BMI *z*-scores using the World Health Organization’s Child Growth Standards^d^ Age range of children in included studies 2–19 years. Mean age 5.61 (SD 2.37) years	Positive All five underlying studies assessed by the authors as being of good quality Adjusts for maternal confounders Negative Only able to use confounders used in most studies Rating: Fair
Aucott 2017^30^	Population-based cohort (linkage) study Scotland *n* = 5863 mothers with more than one linked pregnancy and 718 sibling pairs of children	1. Smoking status^b^Recorded at the start of each pregnancy and categorized: Never smoked, quit, started smoking, always smoked 2. Maternal weight change ^c^Broken down into 7 categories from 10% or greater loss to 10% or greater gain. Reference category ±3.0%.	BMI categories using the International Obesity Task Force criteria for weight categories ^c^ BMI *z*-scores (derived using the 1990 UK standard)^c^ Children mean age 5.6 (SD 0.6) years	Positive Routinely collected population level data for exposure and outcome Objectively measured outcome Adjusts for maternal confounders Excludes mothers >16 weeks pregnant at first antenatal appointment in order to exclude weight gain due to pregnancy Negative Smoking status self-reported at a single point in pregnancy only Maternal and child records only linked in 44% of cases Rating: Good
Barclay 2018^31^	Population-based cohort (linkage) study Sweden *n* = 102 000	Length of the interpregnancy interval^c^ Defined here as the birth-to birth interval	BMI categories using standard cut-offs ^c^ Outcome measurements in men only, aged between 17 and 20 years at conscription Study based on full siblings only and in sibling groups of at least 3 Whole sibling groups used for measures of birth spacing, not just men	Positive Routinely collected population level data for exposure and outcome Objectively measured outcome Sibling groups included only where neither partner has children with a third partner Negative Men only for outcome measurement Based on sibling groups with at least 3 children Only maternal confounder controlled for is age Rating: Good
Devakumar 2016^32^	Prospective cohort Brazil *n* = 2239	Length of the interpregnancy interval^b^ Defined as the period from the birth of the previous chid to the beginning of the pregnancy of the index child	Body composition; fat mass, fat-free mass, visceral fat, subcutaneous fat^c^ BMI^c^ Offspring mean age 30.2 years	Positives 68% follow-up rate at offspring age 30 years Objectively measured outcome Adjusts for maternal confounders Negatives Self-reported birth interval length based on maternal recall Rating: Fair
Huttly 1992^33^	Longitudinal population-based cohort Brazil *n* = 2952	Length of the interpregnancy interval^b^ Here the interval since the preceding birth was recorded and is referred to as the ‘birth interval’	Weight for age and height *z*-scores, standardized to National Centre for health statistics standards^c^ Mean age 19 months	Positives 82% of eligible children have outcome data at mean age 19 months Objectively measured outcome Adjusts for maternal confounders Negatives Self-reported birth interval length based on maternal recall Does not report variance Rating: Fair
Iliadou 2010^34^	Population-based cohort (linkage) study Sweden *n* = 8441 sibling pairs	Smoking status^b^ Recorded at the start of each pregnancy and categorized: non-smoker, smoking 1–9 cigarettes a day, smoking ≥10 cigarettes a day	BMI^c^ Men only aged approximately 18 years at conscription	Positive Routinely collected population level data for outcome Objectively measured outcome Adjusts for maternal confounders Negative Men only for outcome measurement Smoking status self-reported at a single point in pregnancy only Rating: Good
Li 2018^35^	Longitudinal cohort study America *n* = 2119	Length of the interpregnancy interval^c^ Defined as the date of the previous birth to the conception of the subsequent pregnancy	BMI *z*-scores standardized using US national reference data^c^ Children aged 7 years	Positives Objectively measured outcome Adjusts for maternal confounders Negatives Study based on very old data with very young mothers; final child measurements taken in 1972 Total duration from recruitment to last birth only 6 years, limiting length of available birth intervals Rating: Poor
Smithers 2017^36^	Population-based cohort (linkage) study Australia *n* = 4099	Birth by elective caesarean or by vaginal birth in the second pregnancy where the previous birth was by caesarean section^c^	Age and sex-specific BMI *z*-scores using the World Health Organization’s Child Growth Standards^c^ Children aged between 3 and 6 years	Positives Routinely collected population level data for exposure and outcome Objectively measured outcome Adjusts for maternal confounders Negatives No information on low linkage rates Rating: Good
Willmer 2013^37^	Population-based cohort (linkage) study Sweden *n* = 71 sibling pairs	Maternal weight loss due to bariatric surgery between pregnancies^c^	Measurements from child health Centre height and weight recorded at comprehensive check-up at age 4, and then predicted BMI at 4th birthday ^c^ Models also tried with BMIs converted to z-scores and with standardization against a British reference population^c^	Positives Routinely collected population level data for exposure and outcome Objectively measured outcome Negatives Only 71 child–woman triads with complete data available for analysis Limited adjustments for maternal confounders Rating: Fair
Yuan 2016^38^	Prospective cohort America *n* = 15 630	Birth method across successive pregnancies; caesarean or vaginal^c^	BMI for individuals under 18 years calculated using the International Obesity Task Force criteria for weight categories^b^ For individuals over 18 years, obesity calculated using World Health Organization cut-offs^b^ Offspring followed from age 9–14 years through age 20–28 years	Positives Controls for maternal confounders Negatives Self-reported outcome Rating: Fair

Abbreviations: BMI, body mass index (kg/m^2^); SD, standard deviation.

a Quality was assessed using an adapted version of the National Heart, Lung and Blood Institute assessment tool for observational cohort and cross-sectional studies^39^ and the PRISMA-IPD checklist.^41^

b Outcome/exposure based on self-reported information

c Outcome/exposure objectively measured

d Details of whether outcome/exposure measurements were self-reported or objectively measured are not stated

**Table 2:** Associations between interpregnancy change in maternal exposures and childhood adiposity in second or higher order siblings across the 11 included studies

	First author and year										
Maternal exposure	Adane 2018^29^	Albers 2018^40^	Aucott 2017^30^	Barclay 2018^31^	Devakumar 2016^32^	Huttly 1992^33^	Iliadou 2010^34^	Li 2018^35^	Smithers 2017^36^	Willmer 2013^37^	Yuan 2016^38^
Interpregnancy weight change											
● Highest loss category^a^	**x**		**+**								
● Loss	**x**		**x**								
● Small/moderate gains	**x**		**x**								
● Highest gain category^b^	**+**		**+**								
● Change in BMI at week 10 of pregnancy										**x**	
Smoking status											
● Smoking PG2 only			**+**				**x**				
● Smoking PG1 only			**+**				**x**				
● Smoked PG1 and PG2			**+**				**+**				
● Increase in each additional cigarette smoked		**+**									
Mode of birth											
● Elective caesarean compared to vaginal birth after a caesarean birth									**x**		
● Vaginal birth after a caesarean birth											**-**
● Caesarean birth after a vaginal birth											**x**
Length of the interpregnancy interval											
● Longer preceding birth intervals^c^				**+**	**x**	**+**		**x**			
● Shorter preceding birth intervals^d^				**x**		**-**					
● Preceding interval<18 months				**x**							
● Continuous variable					**x**			**x**			
● Continuous variable—Female offspring					**+**						
● Continuous variable—Male offspring					**x**						
● Binary variable^e^ **x**											

Notes: (+) factor associated with greater childhood adiposity; (−) factor associated with reduced childhood adiposity; (x) confidence interval for association includes the null (1.0 for odds and risk ratios, 0.0 for linear coefficients), or *p* values >0.05 where these are unavailable. Abbreviations: BMI, body mass index; PG1, Pregnancy 1; PG2, Pregnancy 2.

a Adane et al. used loss of ≥1 BMI units; Aucott et al. used loss ≥−10%.

b Adane et al. used gain of ≥4 BMI units; Aucott et al. used gain ≥10%.

c Barclay and Kolk 31 months plus; Devakumar et al. 18 months plus; Huttly et al. 24 months plus; Li et al. 6 months plus.

d Barclay and Kolk 19–24 months; Huttly et al. 18–23 months.

e The only association found was with fat-free mass (see Table 3).

**Table 3:** Summary of statistical analysis, adjustments made and significant results reported across the 11 included studies

First author and year	Statistical analysis	Significant results reported*	
		Exposure	Outcome
Adane 2018^29^	Logistic regression	Interpregnancy weight gain (≥ 4 kg/m^2^) (Ref: Stable interpregnancy weight (± 1 kg/m^2^))	Obesity (aOR 2.20, 95% CI [1.02, 4.75])
Adjustments: Maternal characteristics prior to the second birth (age, area of residence, education, smoking and physical activity), interpregnancy interval and BMI prior to the first birth			
Albers 2018^40^	Individual patient data meta-analysis on five studies. Multilevel model separating within-family and between-family effects	Each additional cigarette smoked a day in sibling pregnancies	BMI *z*-score (β = 0.007, 95% CI [0.006, 0.009])
Adjustments: Maternal weight status, breastfeeding and maternal education attained by the start of the respective pregnancy			
Aucott 2017^30^	Multinomial multilevel logistic modelling and a two level multivariate model. Analysis undertaken with and without sibling analysis	Maternal weight loss >10% (Ref: weight change ±3%)	Overweight (aOR 1.3, 95% CI [1.0, 1.6])
			Obesity (aOR 1.9, 95% CI [1.4, 2.7])
		Maternal weight gain ≥10% (Ref: weight change ±3%)	Mean BMI *z*-score (β = 0.13, 95% CI [0.05, 0.20])
		Maternal smoking:	
		With sibling analysis:	
		● Starting to smoke between pregnancies	Mean BMI z-score (β = 0.19, 95% CI [0.01, 0.36])
		● Smoking in both pregnancies	Mean BMI z-score (β = 0.10, 95% CI [0.01, 0.20])
		Without sibling analysis:	
		● Starting to smoke between pregnancies	Mean BMI *z*-score (β = 0.22, 95% CI [0.07, 0.36])
		● Smoking in both pregnancies	Mean BMI *z*-score (β = 0.32, 95% CI [0.24, 0.40])
		● Quitting between pregnancies	Mean BMI *z*-score (β = 0.29, 95% CI [0.18, 0.40])
		Odds ratios (Ref: never smokers)	
		● Starting to smoke between pregnancies	Overweight (aOR 1.5, (95% CI) 1.0, 2.2])
			Obesity (aOR 2.0, 95% CI [1.1, 3.6])
		● Smoking in both pregnancies	Overweight (aOR 1.5, 95% CI [1.2, 2.8]) Obesity (aOR 1.8, 95% CI [1.3, 2.6])
		● Quit between pregnancies	
			Overweight (aOR 1.5, 95% CI [1.0, 2.0])
			Obesity (aOR 1.6, 95% CI [1.0, 2.5])
Adjustments: Child sex, maternal weight category, maternal smoking (for weight change exposure)/percentage weight change (for smoking change exposure), socio-economic status, parity, maternal age and birthweight *z*-score			
Barclay 2018^31^	Analysis: Between-family analysis using linear regression and within-family analysis, using sibling fixed effects	Preceding interval length (Ref: 25–30 months)	
		● Between-family analysis: (months)	Overweight/obesity:
		31–36	(β = 0.012, 95% CI [0.005, 0.019])
		37–42	(β = 0.013, 95% CI [0.005, 0.020])
		43–48	(β = 0.016, 95% CI [0.008, 0.024])
		49–54	(β = 0.018, 95% CI [0.009, 0.027])
		55–60	(β = 0.033, 95% CI [0.023, 0.043])
		61–66	(β = 0.036, 95% CI [0.024, 0.047])
		67–72	(β = 0.042, 95% CI [0.029, 0.055])
		73–78	(β = 0.044, 95% CI [0.030, 0.059])
		79–84	(β = 0.057, 95% CI [0.040, 0.073])
		85–90	(β = 0.057, 95% CI [0.038, 0.076])
		91–96	(β = 0.075, 95% CI [0.052, 0.097])
		97+	(β = 0.084, 95% CI [0.070,
			0.098])
		● Within-family analysis: (months)	Overweight/obesity:
		31–36	(β = 0.014, 95% CI [0.000, 0.028])
		55–60	(β = 0.021, 95% CI [0.002, 0.040])
		67–72	(β = 0.034. 95% CI [0.009, 0.059])
		79–84	(β = 0.062, 95% CI [0.031, 0.093])
		97+	(β = 0.039, 95% CI [0.009, 0.068])
Adjustments: Birth order, maternal age, birth year, sibling group size, age at and year of conscription			
Devakumar 2016^32^	Multivariable linear regression	Birth interval ≥ 18 months (ref: <18 months)	Fat free mass (kg) (β = 1.717, 95% CI [0.242, 3.193])
		Continuous birth interval (females)	Fat free mass (kg) (β = 0.014, 95% CI [0.000, 0.027])
			Visceral fat (cm) (β = 0.004, 95% CI [0.000, 0.007])
Adjustments: Maternal age, education, BMI at the beginning of pregnancy, family income at birth and birth order			
Huttly 1992^33^	ANOVA (*p*-value < 0.001)	Birth interval in months	Mean weight-for-height *z*-scores
		<18	0.07
		18–23	-0.03
		24–35	0.06
		36–47	0.23
		48–71	0.19
		>71	0.27
Adjustments: Maternal income, race, education, cohabitation, age and parity			
Iliadou 2010^34^	Analysis: Logistic regression	Smoking in both male pregnancies (Ref: non-smokers in pregnancy)	Overweight (aOR 1.71, 95% CI [1.39, 2.09])
Adjustments: Maternal age, height, BMI, pregnancy weight gain, maternal and paternal socio-economic category and education, offspring birthweight, head circumference, gestational age, urban living and age at conscription			
Li 2018^35^	Generalized linear regression models	Length of the interpregnancy interval	No significant results reported
Adjustments: Infant gender, maternal race, maternal marriage status, SES, mother’s educational level, smoking status, pregestational/gestational diabetes, pregestational/gestational hypertension (in the continuous model only), gestational age, prepregnancy BMI, weight gain in pregnancy, mode of labour onset, birthweight for gestational age, Apgar score at 5 minutes, birthweight (in the categorical model only), feeding pattern, rapid weight gain in the first year of life, and maternal age at birth			
Smithers 2017^36^	Augmented inverse probability weighted analysis	Mode of birth	No significant results reported
Adjustments: Maternal age, type of antenatal care, number of antenatal visits, medical conditions in pregnancy (asthma, diabetes, hypertension), smoking in pregnancy, gestational age, birthweight for gestational age *z*-scores, maternal partnership status, maternal ethnicity, maternal occupation, neighbourhood-level indicators of socioeconomic disadvantage and remote residence			
Willmer 2013^37^	Fixed-effects regression	Maternal weight change	No significant results reported
Adjustments: Sex of siblings, birth order, mother’s age and smoking in pregnancy			
Yuan 2016^38^	Within-family analysis using conditional logistic regression	Vaginal birth after caesarean birth	Obesity (RR 0.69, 95% CI [0.53, 0.83])
Maternal age at birth, race, prepregnancy BMI group, maternal height, gestational diabetes, pre-eclampsia, pregnancy induced hypertension, child sex, year of birth, gestational age at birth, birth order, birth weight group, prepregnancy smoking and region of residence at birth			

Abbreviations: aOR, adjusted odds ratio; BMI, body mass index (kg/m^2^); CI, confidence interval; RR, relative risk.

* Significant results are those where the confidence interval for association does not include the null (1.0 for odds and risk ratios, 0.0 for linear coefficients), or where *p* values <0.05 if these are unavailable.
